# Strengthening and Toughening of ZG25SiMn2CrB Steel without Tempering Brittleness via Electropulsing Treatment

**DOI:** 10.3390/ma17112480

**Published:** 2024-05-21

**Authors:** Yang Zhao, Xinwei Cui, Yuguang Zhao, Zhihui Zhang, Lijun Shi, Baoyu Zhang, Luquan Ren

**Affiliations:** 1 Key Laboratory of Bionic Engineering, Ministry of Education, Jilin University, Changchun 130022, China; 2 Key Laboratory of Automobile Materials, Ministry of Education and Department of Materials Science and Engineering, Jilin University, Changchun 130022, China; 3 Weihai Institute for Bionic, Jilin University, Weihai 264402, China; 4 Liaoning Academy of Materials, Shenyang 110167, China; 5 FAW Foundry Company Limited, Changchun 130062, China

**Keywords:** electropulsing treatment, low-alloy cast steel, tempering, strengthening and toughening

## Abstract

High-strength low-alloy steels are widely used, but their traditional heat-treatment process is complex, energy-intensive, and makes it difficult to fully exploit the material’s potential. In this paper, the electropulsing processing technology was applied to the quenching and tempering process of ZG25SiMn2CrB steel. Through microstructural characterization and mechanical property testing, the influence of electropulsing on the solid-state phase transition process of annealing steel was systematically studied. The heating process of the specimen with the annealing state (initial state) is the diffusion-type transition. As the discharge time increased, the microstructure gradually transformed from ferrite/pearlitic to slate martensite. Optimal mechanical properties and fine microstructure were achieved after quenching at 500 ms. The steel subjected to rapid tempering with 160 ms electropulsing exhibited good, comprehensive mechanical properties (tensile strength 1609 MPa, yield strength 1401.27 MPa, elongation 11.63%, and hardness 48.68 HRC). These favorable mechanical properties are attributed to the coupled impact of thermal and non-thermal effects induced by high-density pulse current. Specifically, the thermal effect provides the thermodynamic conditions for phase transformation, while the non-thermal effect reduces the nucleation barrier of austenite, which increases the nucleation rate during instantaneous heating, and the following rapid cooling suppresses the growth of austenite grains. Additionally, the fine microstructure prevents the occurrence of temper brittleness.

## 1. Introduction

High-strength low-alloy steels (HSLASs) are widely used in equipment manufacturing, vehicle engineering, architecture, etc. They were developed based on low-carbon steel or ordinary carbon steel. High strength and significant toughness could be achieved through processing techniques such as compositional adjustment, heat treatment, and cold/hot deformation [1,2,3,4]. However, the industrial development requires the high strength and high plasticity of steel. The low-alloy steel via traditional heat treatment has not been satisfactory. 

Researchers have used a variety of methods to solve the difficulty of combining strength and plasticity in low-alloy steels. For example, annealing after large plastic deformation, and the introduction of residual austenite in the martensitic matrix have been tried. The annealing treatment after major plastic deformation significantly refined the grain to improve the strength and plasticity of the material. Zhong et al. [5] prepared a nanocrystalline Al-Zn alloy with a grain size of 15 nm using high-pressure torsion, which has an ultra-high yield strength of about 642 MPa. Zhang et al. [6] proposed a new method of severe plastic deformation called three-dimensional severe plastic deformation. The ferrite matrix in 20MnSiV steel was refined to 3 μm after a single pass of three-dimensional intense plastic deformation at 650 °C. At the same time, the steel obtained excellent mechanical properties (tensile strength: 626.29 MPa and elongation: 20.82%). However large plastic deformations are mainly applied to lower strength steels. High-strength low-carbon alloy steels with a martensitic matrix have higher requirements for severe plastic deformation equipment. Currently the main methods of introducing residual austenite are Quenching-Partitioning (QP) treatments. A QP treatment is a process in which the specimen is heated to the austenite temperature, then quenched between the Ms-Mf temperatures, and then tempered to retain the residual austenite [7,8,9]. QP steel and Quenching-Partitioning-Tempering (Q-P-T) steel, developed based on the QP process concept, are new martensitic steels with high toughness and high plasticity obtained through quenching and carbon partitioning processes based on TRIP steel. The low-carbon lath martensite ensures the strength, while the rich carbon residual austenite induces phase transformation during deformation, thereby improving the steel’s plasticity. The tensile strength of QP steel is 800~1500 MPa, with an elongation of 15%~40% [10]. Conventional QP treatments can improve the plasticity of the material while maintaining a high tensile strength. However, the selection of the quenching temperature for QP treatment affects the stability of the residual austenite, which influences the improvement of mechanical properties [11]. Therefore, it is necessary to find a new heat treatment to improve the strength and plasticity of HSLASs. 

Electropulsing treatment, as a new technology for controlling the material’s microstructure and properties, has the advantages of high-energy density, efficiency, and energy saving, and has attracted widespread attention from researchers. The current induced coupling effects of Joule heating, electron wind impact, electric migration, and electromagnetic effects can significantly affect diffusion, phase transformation, recrystallization, and other behaviors of metal materials during processing. Previous studies have shown that the pulse current has achieved good results in refining the metal solidification microstructure [12], improving plasticity [13,14], accelerating recovery and recrystallization [15], repairing defects [16], and improving fatigue performance and other fields [17,18,19]. It is well known that phase transformation has always been an important process for controlling the microstructure and properties of steel materials, and they exhibit complex and diverse phase transformation behavior. Therefore, studying the phase transformation of low-alloy steel under electric fields and deeply exploring the mechanism of the extreme non-equilibrium process generated by pulse current on microstructure and phase transformation rules will provide important theoretical basis for improving material performance. 

In this study, ZG25SiMn2CrB steel was selected as the experimental material, and the microstructure of the material was controlled by pulsed current quenching and tempering. Excellent mechanical properties were obtained. The mechanism of pulsed current to eliminate temper brittleness was elaborated according to the analysis of microstructure and mechanical properties, and the optimal pulsed current treatment parameters were obtained. In Section 2, we introduce the experimental materials and testing equipment used in this paper. In Section 3, we discuss in detail the effects of pulsed current theory and conventional treatment on the microstructure and mechanical properties of the materials. Finally, in Section 4, we conclude this work. This study provides guidance for the optimization of the mechanical properties of low-alloy steel.

## 2. Experimental Materials and Methods

### 2.1. Alloy Melting

The low-alloy steel ZG25SiMn2CrB used in this study was melted in a laboratory medium-frequency induction furnace for casting. The designed chemical composition (mass fraction, %) was: 0.25C, 2.0Mn, 1.5Si, and 0.5Cr. The chemical composition of the furnace charge is shown in Table 1, and the actual chemical composition of the experimental steel produced is shown in Table 2. The composition of casting samples was measured by spectrometer. The type and manufacturer of the spectrometer can be found in Table 3.

**Figure 1:**
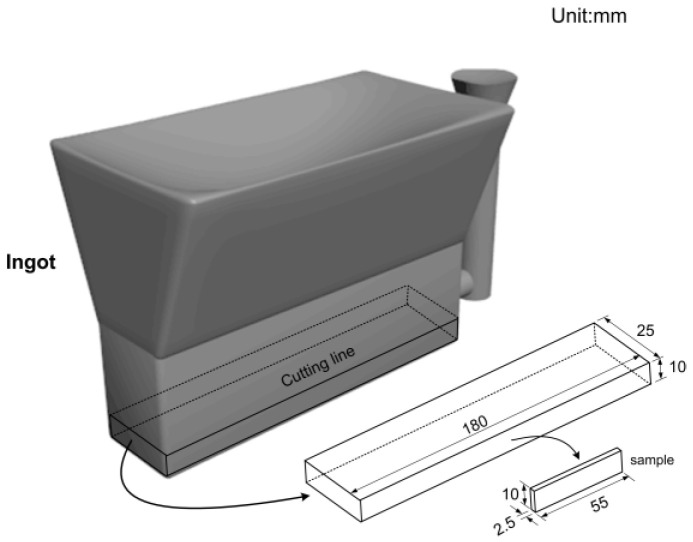
Sketch of the ingot and small samples of ZG25SiMn2CrB steel.

Due to the high susceptibility to oxidation of rare earth, titanium, boron, and other alloying elements, these alloys were preheated and placed into the ladle in advance. The raw materials, including Q235 round steel, high-carbon ferromanganese, medium-carbon ferromanganese, etc., were accurately calculated and weighed before melting in the medium-frequency induction furnace. Aluminum wire was added for deoxidation. Once the desired temperature was reached, the molten steel was poured into resin sand molds to obtain wedge-shaped specimens. After cooling, the sand molds were removed using a grinding machine, and the risers were cut off using a cutting machine (as shown in Figure 1). The white block in Figure 1 shows a homogeneous, non-porous sample cut from the casting sample (dimensions: 180 × 25 × 10 mm). Then, the sample for the tensile experiment was cut from this sample (size: 55 × 10 × 2.5 mm).

### 2.2. Sample Preparation

**Figure 2:**
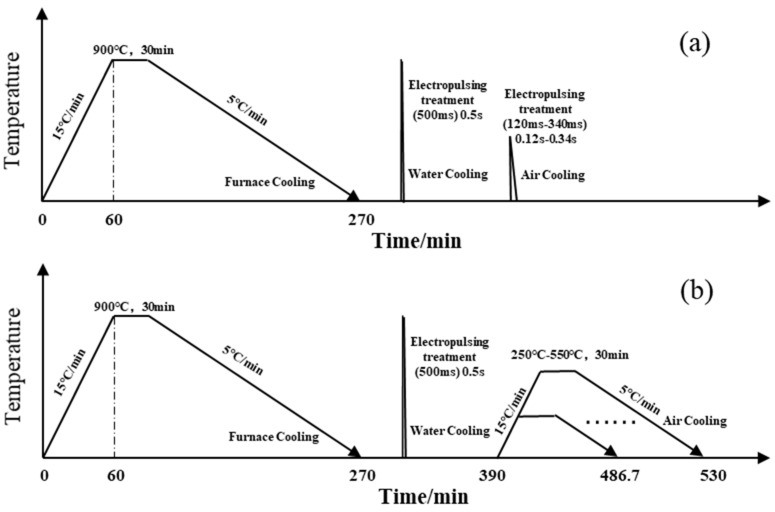
Scheme of the technological process used: (**a**) electropulse quenching + conventional tempering; (**b**) electropulse quenching and tempering.

The specific process of the experiment is illustrated in Figure 2, where the experiment compares the changes in microstructural and mechanical properties between short-term electropulsing tempering and traditional long-term heat-treatment furnace tempering processes. The heat treatment only qualitatively reflects the heat treatment process. In the first stage, the ZG25SiMn2CrB was annealed as a pretreatment. Using an electrical discharge wire-cutting machine, flat plates of dimensions 180 × 25 × 10 mm^3^ were cut from the bottom of the casting. Several of these plates were placed in a vacuum box-type resistance furnace, heated to 900 °C at a rate of 15 °C/min, and maintained at this temperature for 30 min before cooling in the furnace. 

In the second stage, the annealed experimental steel was treated with a 500 ms pulse current followed by water quenching. Finally, the samples processed in the first two stages were divided into two groups. One group underwent traditional heat treatment (furnace tempering) at intervals of 50 °C within the range of 250 °C to 550 °C for 30 min (Figure 2a). The other group underwent rapid tempering with the pulse current starting from 120 ms, increasing in increments of 20 ms up to 340 ms (Figure 2b). Before the electropulsing treatment, the sample surfaces were polished to remove traces left by wire cutting and reduce the contact resistance between the copper electrodes and the samples during the electropulsing treatment.

### 2.3. Electropulsing Treatment Device and Experimental Procedure

**Figure 3:**
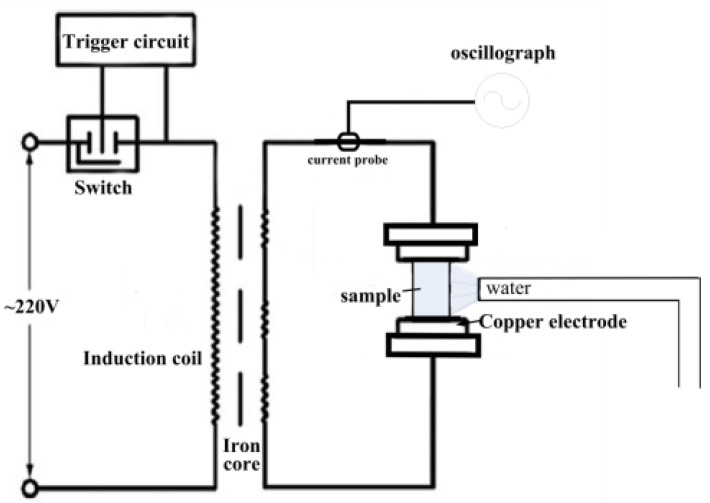
The schematic illustration for the electropulsing system.

The electropulsing treatment device used in this experiment was a 220 V voltage, 50 Hz fixed-frequency power supply. The current, generated by a transformer with primary and secondary induction coils, was converted into a low-voltage power supply. The resulting current belonged to alternating pulse current (refer to the circuit diagram in Figure 3). The applied voltage and processing time are controlled by a specific program in the computer. At the same time, the samples are clamped before treatment in order to minimize contact resistance. The operation of the pulsed current device consists of setting the relevant parameters (voltage, current, treatment time, etc.) in the computer, then turning on the control switch and the sample is programmed to complete the electrical pulse treatment. During the experiment, a MIYACHI (MM-315B, Tokyo, Japan) current meter was used to monitor the equivalent current in real-time within the circuit. The electropulsing treatment process was controlled by a computer connected to the trigger. A self-made water-spray system was placed on both sides of the sample for water cooling. The spray nozzles were directly connected to the water supply, and they were immediately activated until the sample cooled to room temperature.

### 2.4. Microstructure Analysis and Testing

**Figure 4:**
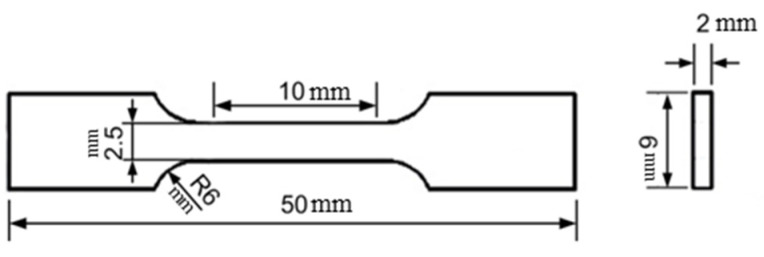
Sketch map of tensile samples.

The specific types and manufacturers of the equipment used in this study are shown in Table 3. The chemical composition of the materials used in the experiment was measured using a Swiss-made ARL4460 direct-reading spectrometer, which had a measurement accuracy of 3–5 ppm. The samples were sequentially pre-ground and mechanically polished. Afterward, the samples were etched with a 4% nitric acid alcohol solution for a certain period at room temperature, followed by rinsing with alcohol and drying. Optical microstructure observation and photography were conducted using a German Zeiss (AxioImager. A2m) metallographic microscope. Scanning electron microscopy (SEM) was used for observing the microstructure and was performed using a ZESIS-EVO18 SEM from Jena, Germany. Tensile tests were carried out at room temperature using an MTS-810 electro-hydraulic servo material-testing machine manufactured in the United States, with a tensile rate set at 0.2 mm/min. The elongation of the samples was measured by a tensiometer. Before testing, the gauge length portion was polished to a bright finish to avoid stress concentration and the influence of cutting marks on the experimental results. The dimensions of the specimens are shown in Figure 4. The R6 represents the arc diameter of 6 mm. Rockwell hardness testing was performed using a 200HRS-150 digital Rockwell hardness tester. Three specimens were taken for each group, and five points were measured on each specimen to obtain the average value as the final hardness value. The specific types and manufacturers of the equipment used in this study are shown in Table 3.

## 3. Experimental Results and Discussion

### 3.1. Microstructure Changes after Traditional Tempering and Electropulsing Tempering

Casting microstructure tends to exhibit severe dendritic segregation, coarse columnar grains, equiaxed grains, and Widmanstätten structures, which is highly heterogeneous. Additionally, differences in casting structure and wall thickness can lead to variations in solidification rates and microstructure, resulting in significant residual casting stresses. Therefore, a pre-annealing treatment was always applied to the cast steel, involving heating it to 900 °C, then furnace-cooling it to 500 °C, and subsequently air-cooling it to ensure uniformity in the microstructure and composition.

**Figure 5:**
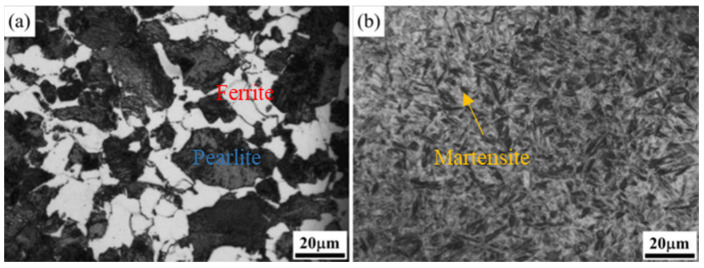
Microstructure of the experimental steel: (**a**) 900 °C annealing pretreatment; (**b**) 500 ms electropulsing quenching after the annealing.

The microstructure of the cast steel after the annealing pretreatment is shown in Figure 5a, mainly consisting of ferrite (white) and pearlite (gray-black). After the annealing pretreatment, the samples underwent 500 ms electropulsing treatment and water quenching to achieve complete austenitization and uniform fine-quenched martensite, as shown in Figure 5b. As is known, the lath martensite substructure in low-alloy steel consists of dislocations, which contribute to its high strength and hardness.

### 3.2. Influence of Traditional Tempering on Microstructure of Electropulsing Quenched Samples

**Figure 6:**
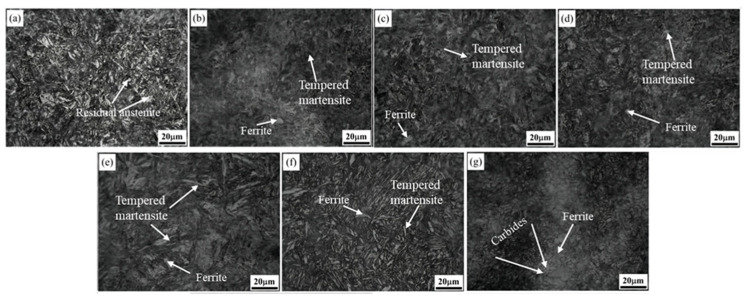
Microstructures of experimental steel after conventional tempering at different temperatures (OM): (**a**) 250 °C; (**b**) 300 °C; (**c**) 350 °C; (**d**) 400 °C; (**e**) 450 °C; (**f**) 500 °C; (**g**) 550 °C.

The evolution of the microstructure after traditional tempering from 250 °C to 550 °C for the quenched steel is illustrated in Figure 6. Tempering is an effective means of reducing the brittleness of martensite by supersaturated carbon atoms precipitating, thereby obtaining better comprehensive mechanical properties. Under traditional tempering processes, the low-alloy steel after quenching undergoes several changes:

- In the temperature range of 80 °C to 200 °C, the precipitation and redistribution of transition carbides occur at dislocations, lath boundaries, and grain boundaries. ε-carbides (ε-Fe_2.4_C) also form during this stage, known as the first stage of tempering.
- With the increasing temperature (200 °C to 300 °C), the thin-film residual austenite decomposes into α-ferrite and θ-carbides (θ-Fe_3_C), entering the second stage of tempering.
- When the temperature exceeds 300 °C, the metastable carbides transform into stable carbides (Fe_3_C) in the third stage of tempering, which completes before 450 °C. After tempering temperatures surpass 350 °C, cementite undergoes spheroidization and coarsening.

**Figure 7:**
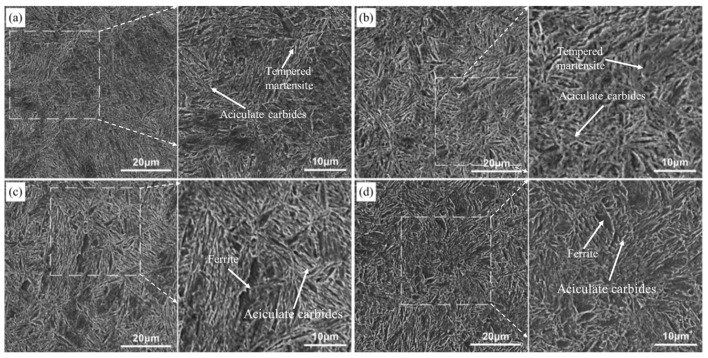
Microstructures of experimental steel after conventional tempering at different temperatures (SEM): (**a**) 250 °C; (**b**) 350 °C; (**c**) 450 °C; (**d**) 550 °C.

Figure 7 shows the image corresponding to tempering at 250 °C, 350 °C, 450 °C, and 550 °C, measured with SEM. The white dashed box area in Figure 7a–c corresponds to the right-hand side picture. The microstructure of the steel after electropulse quenching and traditional low-temperature tempering at 250 °C is mainly a tempered martensitic structure (Figure 6a), part of the martensitic lath bundles are polygonal, and fine aciculate carbides precipitate preferentially along the boundary of the martensitic lath bundles (Figure 7a). As is known, after the traditional tempering heat-treatment, the dislocation density of the martensite in the steel gradually decreases, the boundary of the strip becomes blurred, and the contrast between the tempered martensite interface and the microstructure inside the grains is more obvious than in the quenched state, indicating that the diffusion of carbon atoms and interstitial elements and the decomposition of martensite during the tempering process make the phase interface and the intragranular composition vary. As the tempering temperature increased from 300 °C to 550 °C (Figure 6b–g), the recovery of martensitic became more obvious, the phase interface gradually became blurred, the martensitic swallowed and was merged, and the internal substructure became coarser. The substructural unit is formed between the laths, and the microstructure is more uniform, so it has excellent plasticity, which shows that the plasticity increases and the tensile strength decreases (the mechanical property change will be discussed below). After tempering at 550 °C, parts of the steel recrystallized to form a quasi-polygonal ferrite, as shown in Figure 6g. These processes are carried out spontaneously with increasing temperature and time, accompanied by energy release, and the driving force is the free enthalpy difference between steady and metastable states [20]. The microstructure evolution process from quenched martensite to traditional tempering treatment at different temperatures is mainly the recovery of lath martensite and the dislocation substructure, residual austenite decomposition, and recrystallization softening process [21]. With the increase in the tempering temperature in the heat treatment furnace, the residual austenite content in steel decreases continuously, and carbides precipitate in the martensite. As the carbon element diffuses from martensite to austenite, the carbon concentration of martensite decreases, leading to constant changes in carbide morphology. Additionally, precipitation occurs, making the microstructure more susceptible to etching, thereby enhancing the contrast (Figure 6).

### 3.3. Influence of Electropulsing Tempering on Microstructure of Electropulsing Quenched Samples

**Figure 8:**
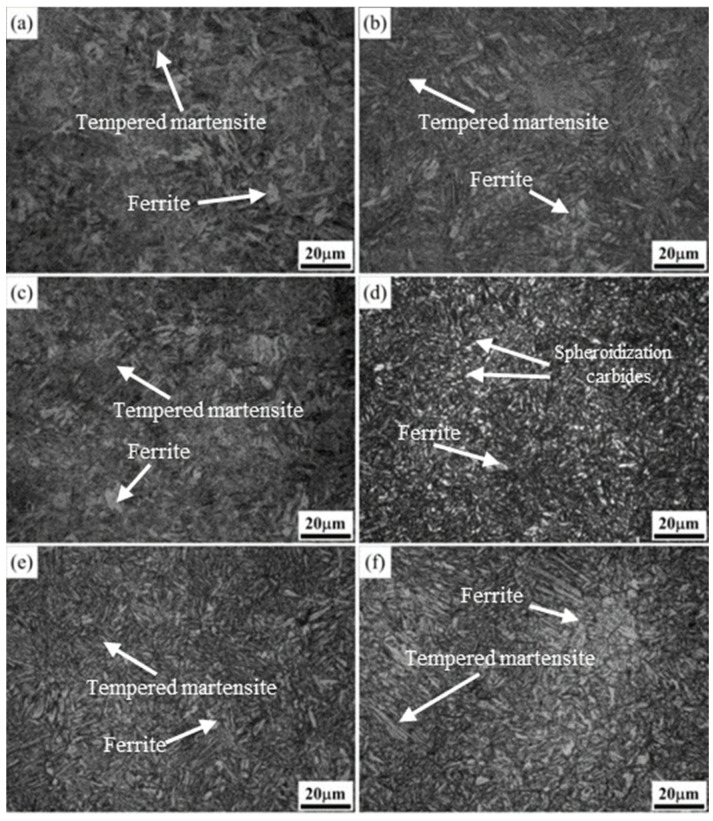
Microstructures of experimental steel after electropulsing tempering at different times (OM): (**a**) 120 ms; (**b**) 140 ms; (**c**) 160 ms; (**d**) 180 ms; (**e**) 200 ms; (**f**) 220 ms.

**Figure 9:**
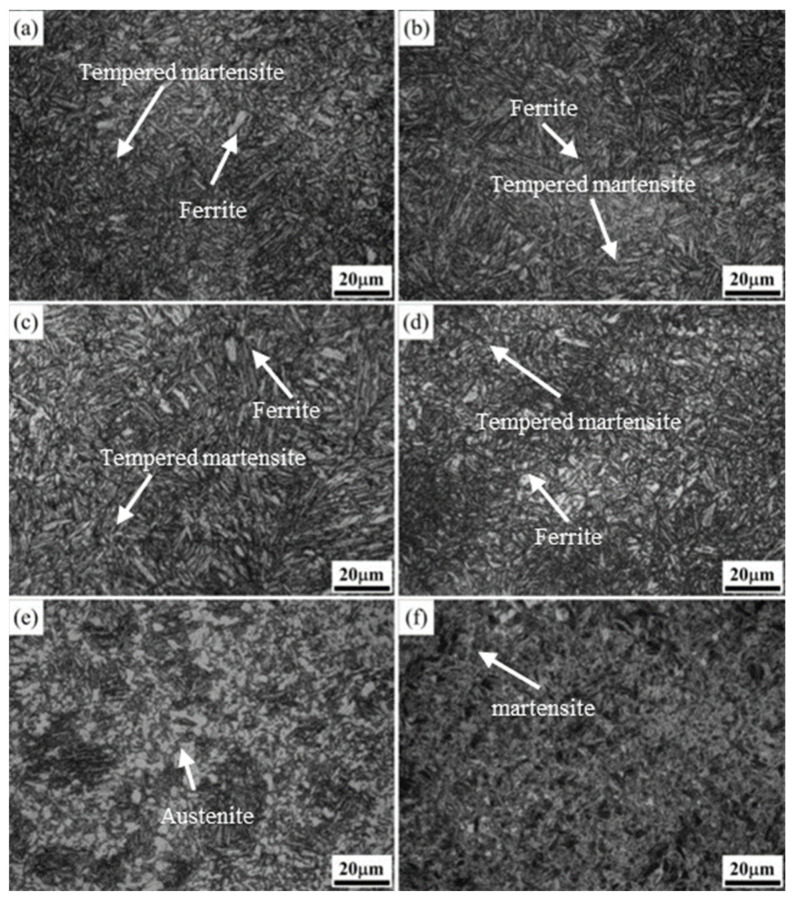
Microstructures of experimental steel after electropulsing tempering at different times (OM): (**a**) 240 ms; (**b**) 260 ms; (**c**) 280 ms; (**d**) 300 ms; (**e**) 320 ms; (**f**) 340 ms.

**Figure 10:**
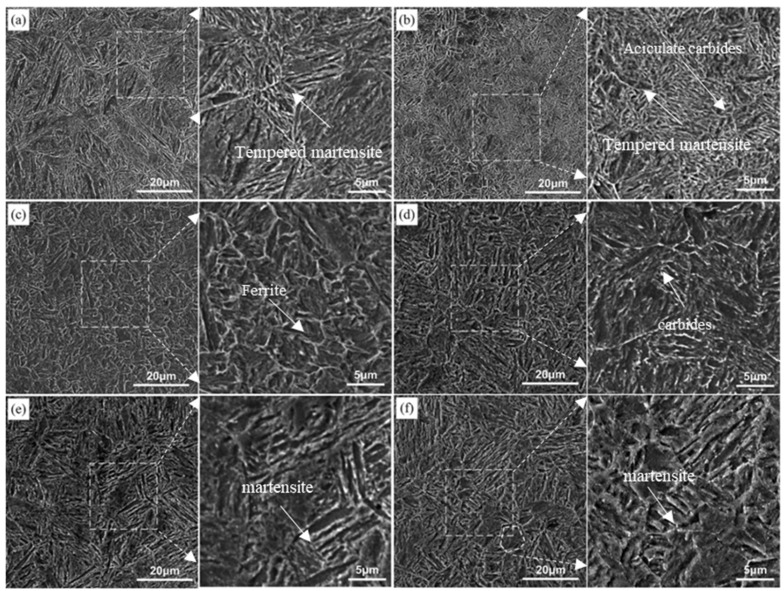
Microstructures of experimental steel after electropulsing tempering at different times (SEM): (**a**) 120 ms; (**b**) 160 ms; (**c**) 180 ms; (**d**) 220 ms; (**e**) 260 ms; (**f**) 300 ms.

Figure 8 and Figure 9 illustrate the evolution of the microstructure for the quenched steel after electropulsing tempering from 120 ms to 340 ms, while Figure 10 shows the corresponding SEM images of tempering conditions. Figure 8a displays the microstructure after 120 ms of electropulsing tempering. Figure 10 shows the measurements with a scanning electron microscope (SEM). The white dashed box area in Figure 10a–f corresponds to the right-hand side of the picture. The SEM image in Figure 10a shows the carbide precipitation within the laths, but less than observed in the samples subjected to traditional low-temperature tempering (Figure 7a). The microstructure after 140 ms electropulsing tempering, as shown in Figure 8b, mainly consists of tempered martensite laths with some ferrite. After 160 ms electropulsing (Figure 10b), the lath becomes finer. In Figure 10c, when the electropulsing time reaches 180 ms, there is a further reduction in the fine carbides, and their morphology becomes spheroidized, resulting in a finer microstructure, indicating that the electric field promotes the spheroidization of carbides. Nan-lin Wang [22] and others also observed this phenomenon in their study on the effect of an electric field on medium-carbon-quenched steel. It was indicated that carbon atoms within the grain preferentially combine with vacancies to form a combination of solute atoms and vacancies. The interaction between the vacancies and charges promotes the migration of vacancies, accelerating the diffusion of carbon atoms and promoting the segregation and spheroidization of carbides. The magnified image is shown in Figure 10c, and the directionality of the microstructure is also reduced. With the electropulsing time exceeding 200 ms, as shown in Figure 8f, the microstructure appears coarser compared to 180 ms and 160 ms tempering. The lath martensite structure remains retained, which is beneficial for maintaining the mechanical properties. From Figure 9c,d, it can be observed that after 280 ms and 300 ms of electropulsing tempering, recrystallization is less apparent compared to traditional tempering. The athermal effect of electropulsing promotes grain refinement due to the increased nucleation rates [13]. However, the short duration of the pulse and the rapid cooling hinder the growth of recrystallized grains, leading to finer microstructures and enhanced strength and ductility. From the distribution of metallographic colors, it can be seen that the color distribution after electropulsing tempering is more uniform, also indicating a more uniform distribution of carbon. However, the recovery process causes the lath martensite structure to merge, resulting in wider sizes and clearer blocky grain boundaries.

When a 320 ms electropulsing treatment is applied, the temperature of the experimental steel rises again to between Ac_1_ and Ac_3_, and after cooling, an incompletely transformed austenitic microstructure is obtained, as shown in Figure 9e. In this case, fine austenitic grains form a net shape along the original austenite grain boundaries, dividing the large untransformed austenite into small islands. After 340 ms of electropulsing treatment, the temperature of the steel rises above Ac_3_, resulting in a reformation of the martensitic structure, as shown in Figure 9f.

### 3.4. Mechanical Properties of Quenched Samples Treated by Traditional Tempering and Electropulsing Tempering 

**Figure 11:**
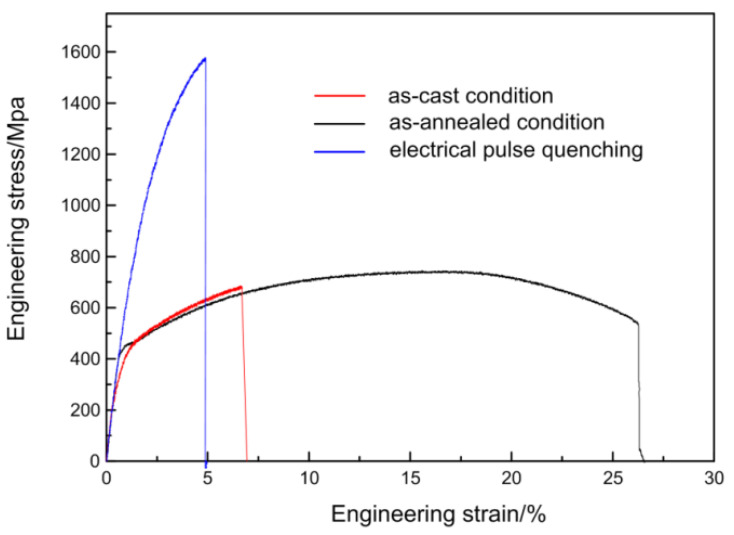
Tensile engineering stress–strain curves of experimental steel after homogenization annealing, electrical pulse quenching, and as-cast.

Figure 11 depicts the mechanical performance of ZG25SiMn2CrB in its as-cast state, annealing at 900 °C, and 500 ms of electropulsing quenching. The as-cast samples exhibit poor mechanical properties owing to their coarse and uneven microstructure After annealing, the steel exhibits a significant increase in elongation but a lower strength. After electropulsing quenching, the steel achieves a yield strength of 944 MPa and a tensile strength of 1575 MPa, demonstrating high strength which is due to the fine martensitic microstructure obtained by pulsed current quenching, but the martensitic matrix is brittle and therefore the material has a low elongation (5%).

#### 3.4.1. Mechanical Properties of Quenched Samples Treated by Traditional Tempering 

**Figure 12:**
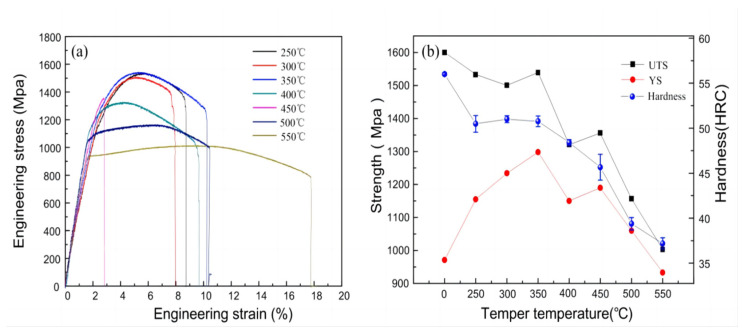
Mechanical properties of experimental steel after conventional tempering at different temperatures. (**a**) Engineering stress-strain curves; (**b**) Strength and hardness variation curves.

After quenching, the samples were subjected to traditional tempering heat-treatment at different temperatures for 30 min (Figure 12 and Table 4). It was found that both the tensile strength and Rockwell hardness decreased, while the elongation increased. The yield strength initially increased and then decreased with the increase in tempering temperature, reaching its peak at 350 °C. The main reasons for strengthening were the results of a combination of dislocation strengthening, solid solution strengthening, grain refinement strengthening, and dispersion strengthening. At low-temperature tempering (250 °C), the recovery phenomenon was not significant. The main strengthening mechanism was the decomposition and precipitation of residual austenite. Tempering treatments below 350 °C eliminated some of the residual stresses produced by quenching, while improving the uniformity of the microstructure, resulting in improved plasticity and toughness compared to the quenched state. On the other hand, the residual austenite decomposes into martensite and ε-carbides during tempering led to a slight softening effect due to recovery, which was less than the strengthening effect of precipitation hardening, resulting in an increase in yield strength but a decrease in tensile strength. As the temperature gradually increased, the recovery phenomenon became obvious. The non-equilibrium lath martensite structure gradually evolved towards equilibrium, with lath boundaries widening through merging and mutual engulfment, and dislocation defects gradually disappearing, significantly affecting the tensile strength. An anomaly occurred around 300 °C and 450 °C, where both the tensile strength and elongation sharply decreased. The samples experienced low-stress brittle fractures before the yield stage, the phenomenon known as temper embrittlement, observed in quenched martensitic steels tempered between 250 °C and 500 °C. In this experiment, the traditional tempering process led to temper embrittlement, possibly due to the coarsening of ε-carbides with increasing temperature. Although the steel contained around 1.4% silicon, which inhibits the transformation of ε-carbides to Fe_3_C, fine-dispersed ε-carbides did not significantly reduce the mechanical properties of the material. However, as the traditional tempering temperature increased, ε-carbides gradually coarsened, and the coarse ε-carbides were the direct cause of the first type of temper embrittlement observed in the steel. Intergranular fractures caused by phosphorus segregation at the original austenite grain boundaries and transgranular fractures caused by the coarse ε-carbides between the thick plates of martensitic bundles were both related to temper embrittlement.

The contributions of the recovery and precipitation phenomena during tempering to mechanical properties are contradictory, and their effects can lead to non-monotonic changes in the mechanical properties, as shown in Figure 12. The yield strength initially increases and then decreases. Meanwhile, plasticity and toughness exhibit a complex trend of initially increasing, then decreasing, and then increasing again. In summary, the changes in mechanical properties after traditional tempering can be attributed to the combined effects of the decomposition of residual austenite and the precipitation of oversaturated carbon in martensite, resulting in second-phase strengthening.

#### 3.4.2. Mechanical Properties of Quenched Samples Treated by Electropulsing Tempering

**Figure 13:**
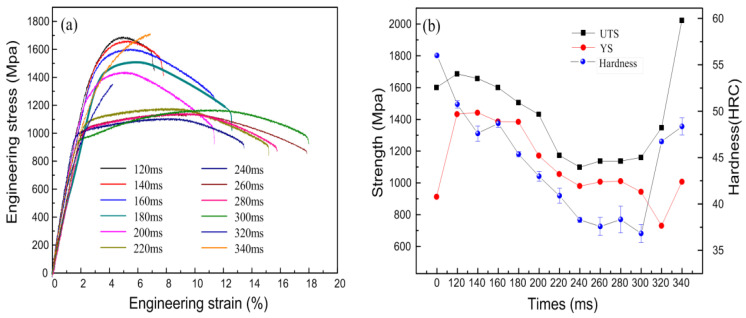
Mechanical properties of experimental steel after electropulsing tempering at different times. (**a**) Engineering stress-strain curves; (**b**) Strength and hardness variation curves.

The tensile curves after rapid tempering with different pulse current duration are shown in Figure 13a. Initially, the tensile strength after tempering is higher than that of the electropulsing quenched state (Table 5). This may be due to the high internal stress of the quenched sample, leading to premature brittle fractures during tensile testing, resulting in a lower ultimate tensile strength. The decreasing trend in mechanical properties from 120 ms to 200 ms is slower compared to traditional heat treatment, and the overall trend of strength remains higher. This indicates that lower-duration electropulsing treatment can reduce the defect density while maintaining the strength and hardness of the quenched state, allowing the residual stress to be released. One of the main reasons for the effect of rapid electropulsing tempering is that the sample temperature increases rapidly, causing highly concentrated instantaneous thermal-compressing stress and electron wind force. As the electropulsing time increases, the peak temperature of the sample gradually increases, leading to recovery and recrystallization phenomena. From 120 ms to 240 ms, when the temperature of the electropulsing treatment is relatively low, the elongation of the material shows an increasing trend, while the tensile strength gradually decreases. The trend of yield strength is consistent with that of tensile strength. Between 120 ms and 200 ms of electropulsing tempering, only point defects and dislocation migrations occur, without notable changes in the grain size and shape. However, the internal stress is greatly reduced. Between 200 ms and 240 ms of electropulsing tempering, the tensile strength decreases significantly. One reason is that as the pulse duration lengthens, the peak temperature increases, causing carbon atoms in the martensite to continuously migrate to the residual austenite, reducing the carbon concentration in the martensite, weakening the solid-solution strengthening effect. Another reason is that as the duration increases, the dislocation density in the lath martensite decreases, weakening the effects of strain hardening and dislocation strengthening. Although there is some fluctuation in tensile strength and yield strength between 240 ms and 280 ms, the change is not significant. From 260 ms to 300 ms, the yield strength gradually decreases, the elongation increases, and the tensile strength does not change significantly. However, at 320 ms and 340 ms, the tensile strength sharply increases. This is because when treated with 320 ms of electropulsing, the peak temperature is too high, and the temperature of the sample rises again to between Ac_1_ and Ac_3_, resulting in the partial austenitization of the microstructure. This partially reversed austenite will transform into martensite during cooling, significantly increasing the hard phase structure, leading to a sharp increase in tensile strength. After cooling, the microstructure obtained is an incomplete austenitic transformation, but the austenite is not retained in the form of residual austenite, resulting in a significant decrease in elongation. However, when treated with 340 ms of electropulsing, the steel temperature rises above Ac_3_, and the martensite structure is reformed after cooling; then, the mechanical properties would be affected. Therefore, the good tempering interval is between 120 ms and 300 ms. Under the combined action of the Joule heat, instantaneous thermal compressing stress, and electron wind force generated by electropulsing tempering, the movement of obstructed dislocation groups is promoted, leading to the orderly opening of entangled dislocations and the gradual release of residual stress inside the sample through microscopic plastic deformation [23]. This configuration reduces the energy of dislocations and increases stability, exhibiting strong resistance to tempering softening.

During the initial heating stage of the traditional tempering treatment (250 °C to 350 °C) and when the electropulsing tempering time is short (120 ms to 180 ms), the changes in mechanical properties are slow, which is related to the slow disappearance of defects such as vacancies and dislocations. While in the later stage of electropulsing tempering (240 ms to 300 ms), the changes in mechanical properties slow down again. It should be noted that the later stage process requires a passage through a time range from 180 ms to 240 ms. During this stage (180 ms to 240 ms), dislocation movement and recovery increase, simultaneously consuming the previously stored energy, reducing the recrystallization driving force, and when the duration exceeds this stage, electropulsing only promotes grain growth through thermodynamic driving force, resulting in a slow recrystallization. In the rapid electropulsing tempering process, unlike traditional tempering, the morphology of grains does not change during the “recovery” process, and the dislocation density remains high.

Cementite (Fe_3_C) is a brittle and hard strengthening phase. However, if it is distributed at grain boundaries, especially in a continuous net shape, it can lead to a significant decrease in plasticity and toughness. In this study, the temper embrittlement phenomenon was not observed during the electropulsing tempering process. Compared with short-term electropulsing tempering, long-term tempering will cause recovery of the matrix, a reduction in the dislocation density, and softening of the strength. By using electropulsing to perform rapid tempering, the process of segregation can be suppressed. Through rapid heating, the thermal diffusion causing embrittlement cannot develop, and Fe_3_C precipitates in a spherical shape, thereby reducing its harmfulness to grain boundaries. On one hand, after electropulsing tempering, the martensite lath morphology can still be maintained, and there are more grain boundaries than traditional tempering heat-treatment, which makes impurities such as P and S distribute uniformly on the unit grain boundary area, mitigating phosphorus segregation and reducing temper embrittlement tendency. On the other hand, there may also be grain boundary effects, effectively dividing fracture units at the boundaries of original fine-grained austenite grains and between martensite lath groups, disrupting the continuity of carbonization films precipitated along martensite laths, boundaries, and subgrain boundaries during tempering, preventing cracks from crossing martensite lath boundaries, and playing a significant toughening role [24].

In terms of comprehensive performance, the strength-elongation product of samples treated at 280 ms and 300 ms is relatively large, at 20,340.93 MPa·% and 20,804.05 MPa·%, respectively, while that of samples treated at 160 ms is 18,696.58 MPa·%. The tensile strength is as high as 1609 MPa, and compared with the traditional heat-treated experimental steel, the strength-elongation product is higher, indicating better mechanical properties.

## 4. Conclusions

In this study, fine microstructures were obtained by pulsed current instead of conventional heat treatment. By selecting suitable pulse current parameters, good comprehensive mechanical properties were obtained (tensile strength 1609 MPa, yield strength 1401.27 MPa, elongation 11.63%, and hardness 48.68 HRC). The conclusions of this study are as follows.

1. The process of austenite transformation during heating from the annealed state is diffusion-controlled. After rapid cooling to room temperature, the microstructure changes from ferrite/pearlite to lath martensite. The optimal electropulsing quenching parameter is 500 ms under the experimental conditions. During the electric pulse tempering process, the tensile strength and yield strength decreased continuously with the increase in tempering time, while the elongation showed a gradual increase with a longer tempering time. After quenching and subsequent 160 ms electropulsing tempering, the mechanical properties could reach a tensile strength of 1609 MPa, a yield strength of 1401.27 MPa, elongation of 11.62%, and Rockwell hardness of 48.68 HRC. After conventional tempering at 450 °C, the morphology of ε-carbides precipitated within the martensitic laths becomes significantly coarser compared to the tempering at 250 °C, resulting in a noticeable decrease in ductility and the occurrence of temper embrittlement. No significant reduction in ductility was observed after electropulsing tempering, indicating that electropulsing can effectively prevent the occurrence of temper embrittlement, which is attributed to the fine microstructure and uniform element distribution. The significant multi-field coupling effect of electropulsing processing refines the microstructure, particularly the non-thermal effects, which can reduce the nucleation barrier of austenite. The instantaneous heating process can improve nucleation rates and promote nucleation transformation, inhibiting grain growth and ultimately refining the microstructure.

## Figures and Tables

**Table 1:** Chemical compositions of the charging materials used for producing ZG25SiMn2CrB steel (wt.%).

Charging Materials	C	Si	Mn	Cr	B	V	Ti	RE	S	P	Fe
Cr-Fe	2	2.5		65					0.025	0.03	Bal.
Mid-carbon Mn-Fe	1.9	2	75.6						0.03	0.3	Bal.
High carbon Mn-Fe	6.7	1.5	65.2						0.03	0.211	Bal.
Si-Fe	0.1	75	0.4	0.3					0.005	0.025	Bal.
V-Fe	0.4	2	0.5			50					Bal.
Ti-Fe	0.1	4.5	2.5				30				Bal.
RE								30			Bal.
B-Fe	0.5	2	0.5		17						Bal.
Q235	0.18	0.22	0.45						0.029	0.025	Bal.

**Table 2:** The chemical composition of experimental steel (wt.%).

Material	C	Si	Mn	Cr	B	P, S	Ti, V, RE
ZG25SiMn2CrB	0.25	1.41	2.07	0.52	0.004	≤0.03	0.05 each
Material	C	Si	Mn	Cr	B	P, S	Ti, V, RE

**Table 3:** Experimental apparatus and equipment.

Instrument Name	UNIT TYPE	Manufacturer
Medium-frequency induction furnace	YFL-50	Zhengzhou FulunYing, Zhengzhou, China
Direct-reading spectrometer	ARL4460	Thermo Fisher Scientific, Waltham, MA, USA
Digital Lowell hardness meter	200HRS-150	Jinan Fangyuan, Jinan, China
Optical microscope (OM)	Zeiss (AxioImager.A2m)	Zeiss AG, Jena, Germany
Scanning electron microscope	1000-B	Hitachi, Japan
Pre-mill machine	YM-1	Jinan Fangyuan, Jinan, China
Abrasive finishing machine	PG-1A	Shennuo Instrument Co., Ltd., Shenzhen, China.
Chamber electric furnace	GWL-1600	Guoju, Luoyang, China
EDM cutting machine tool	MNC-A1	AgieCharmilles, Meyrin, Switzerland
MTS810 Electro-hydraulic servo experimental system	MTS810.22M	MTS Systems, Eden Prairie, MN, America

**Table 4:** Integrated mechanical properties of experimental steels after conventional tempering at different temperatures.

Temper Temperature /°C	Yield Strength /MPa	Tensile Strength /MPa	Elongation /%	Product of Strength and Elongation /(MPa·%)	Hardness /HRC
250	1155 ± 23	1600 ± 26	8.72 ± 0.6	13,952.00	50.48
300	1234 ± 35	1533 ± 16	7.92 ± 1.1	12,141.36	51.00
350	1298 ± 22	1501 ± 42	10.21 ± 0.8	15,325.21	50.76
400	1150 ± 17	1321 ± 35	9.64 ± 0.5	12,734.44	48.40
450	1190 ± 41	1356 ± 11	2.78 ± 0.3	3769.68	45.66
500	1060 ± 32	1157 ± 27	10.43 ± 1.7	12,067.51	39.38
550	933 ± 23	1003 ± 24	17.80 ± 0.9	17,853.40	37.18

**Table 5:** Integrated mechanical properties of experimental steels after electropulsing tempering at different times.

Temper Times /ms	Yield Strength /MPa	Tensile Strength /MPa	Elongation /%	Product of Strength and Elongation /(MPa·%)	Hardness /HRC
120	1254 ± 28	1686 ± 22	7.10 ± 0.3	11,970.60	50.74
140	1398 ± 36	1656 ± 31	7.79 ± 1.2	12,900.24	47.6
160	1401 ± 14	1609 ± 21	11.62 ± 0.5	18,696.58	48.68
180	1390 ± 19	1491 ± 13	12.36 ± 0.6	18,428.76	45.36
200	1185 ± 44	1432 ± 36	11.37 ± 1.1	16,281.84	42.98
220	1057 ± 43	1174 ± 32	15.13 ± 0.8	17,762.62	40.88
240	979 ± 21	1099 ± 18	13.44 ± 0.4	14,770.56	38.28
260	1016 ± 13	1138 ± 11	15.82 ± 0.7	18,003.16	38.325
280	980 ± 32	1137 ± 28	17.89 ± 1.6	20,340.93	37.58
300	947 ± 25	1159 ± 41	17.95 ± 1.3	20,804.05	36.82

## Data Availability

The data presented in this study are available on request from the corresponding author. The data are not publicly available due to technical or time limitations.

## References

[1] Shi H.W. (2013). China Iron and Steel Industry Yearbook.

[2] Wang P., Jiang Z., Geng X., Hao S., Zhang X. (2014). Quantification of Chinese steel cycle flow: Historical status and future options. Resour. Conserv. Recycl..

[3] Weng Y.Q., Yang C.F., Shang C.J. (2011). Development status and trend of low alloy steels in China. Iron Steel.

[4] Janjušević Z., Gulišija Z., Mihailović M., Patarić A. (2014). Effect of Tempering on Mechanical Properties and Microstructure of a High-Strength Low-Alloy Steel. Met. Sci. Heat Treat..

[5] Zhong Y., Zhang B., Fang L., Chen J., Xu W., Li X. (2023). Giant hardening and formation of nanograined supersaturated solid solution in Al-Zn system, Mater. Res. Lett..

[6] Zhang Z., Liu D., Wang Y., Pang Y., Zhang F., Yang Y., Wang J. (2020). A novel method for preparing bulk ultrafine-grained material: Three dimensional severe plastic deformation. Mater. Lett..

[7] Bai S.B., Chen Y.A., Sheng J., Li D.Z., Lu H.H., Bai P.K., Huang Z.Q., Li J.Y., Zhao C. (2023). A comprehensive overview of high strength and toughness steels for automobile based on QP process. J. Mater. Res. Technol..

[8] Gao P.F., Li F., An K., Zhao Z.Z., Chu X.H., Cui H. (2022). Microstructure and deformation mechanism of Si-strengthened intercritically annealed quenching and partitioning steels. Mater. Charact..

[9] Cooman D., Bruno C., Estrin Y., Kim S.K. (2018). Twinning-induced plasticity (TWIP) steels. Acta Mater..

[10] Jiang H.T., Tang D., Mi Z.L., Zhuang B.-T. (2011). Effect of partitioning parameters on the retained austenite in low-carbon Q&P steel. Mater. Sci. Technol..

[11] Wang X., Xu Y.B., Wang Y., Li J.Y., Wang Y., Gu X.L., Misra R.D.K. (2022). Combined effect of Cu partitioning and nano-size precipitates on improving strength-ductility balance of Cu bearing Q&P steel. Mater. Charact..

[12] Ma R., Zhang X.F. (2022). Improvement of mechanical properties and microstructural refining of cast titanium alloys by coupling of electropulsing and temporary alloying element hydrogen. Mater. Sci. Eng. A.

[13] Zhao Y.G., Ma B.D., Guo H.C., Ma J., Yang Q., Song J. (2012). Electropulsing strengthened 2GPa boron steel with good ductility. Mater. Des..

[14] Xiao A., Yan Z.Q., Huang C.Q., Yu Z., Wang S., Cui X. (2023). Reduction of springback of Ti6Al4V alloy by high-density and instantaneous pulsed current. Mater. Sci. Eng. A.

[15] Wu Z.C., Xu X.F., Zhao Y., Yan X., Zhou Y., Wei L., Yu Y. (2023). Investigation of accelerated recrystallization behavior via electropulsing treatment in CoCrFeMnNi high-entropy alloy. Mater. Sci. Eng. A.

[16] Yan X.D., Xu X.F., Wu C., Zhao Y., Li D., Zhou Y., Wei L. (2023). A novel electropulsing treatment to improve the surface strength and repair the pore of additively manufactured Ti-6Al-4V alloy. Surf. Coat. Technol..

[17] Qin S.Y., Ba X., Zhang X.F. (2020). Accelerated cluster dissolution using electropulsing for ultrafast performance regeneration. Scr. Mater..

[18] Wei L., Xu X.F., Zhao Y., Yan X., Zhou Y., Wu Z., Yu Y. (2023). High Thermal Stability of a Colony and Basket-Weave Mixed Microstructure in Selective-Laser-Melted Ti-6Al-4V Alloy Induced by Electropulsing. Metals.

[19] Wei L., Xu X.F., Zhao Y., Yan X., Wu Z., Wu C. (2023). A shortened process of tri-modal microstructure developing in Ti-6Al-4V alloy via electropulsing-induces grain spheroidization. Mater. Charact..

[20] Yu D.G. (2008). Iron-Based Martensite Aging-Tempering Transition Theory and Its Strengthening Toughness.

[21] Wang L.J., Ca Q.W., Wu H.B., Li Y., Lv D., Shi J., Fan J. (2010). Effect of tempering temperature on the microstructure and property of 1500 MPa Direct quenching steel. J. Eng. Sci..

[22] Wang N., Liu W., Wu F., Zhou H., Zhang X., Tang G., Zhu J. (2001). Influence of an electric field on the quench aging of a medium-carbon alloy steel. Scr. Mater..

[23] Zheng J.Y. (2011). Theory and Key Technology of Residual Stress Elimination by Electric Pulse Method.

[24] Zeng Y.Z. (1983). Effect of intercritical quenching on the reversible tempering brittleness for 30CrMnSiA steel. Met. Heat Treat..

